# Surface Modification of Hollow Structure TiO_2_ Nanospheres for Enhanced Photocatalytic Hydrogen Evolution

**DOI:** 10.3390/nano13050926

**Published:** 2023-03-03

**Authors:** Gaomin Ning, Yan Zhang, Chunjing Shi, Chen Zhao, Mengmeng Liu, Fangfang Chang, Wenlong Gao, Sheng Ye, Jian Liu, Jing Zhang

**Affiliations:** 1 School of New Energy, Nanjing University of Science and Technology, Fuxing Road 8, Jiangyin 214000, China; 2 College of Science & School of Plant Protection, Anhui Agricultural University, Hefei 230036, China; 3 State Key Laboratory of Catalysis, Dalian Institute of Chemical Physics, Chinese Academy of Sciences, 457 Zhongshan Road, Dalian 116023, China; 4 Dalian National Laboratory for Clean Energy, 457 Zhongshan Road, Dalian 116023, China; 5 College of Chemistry and Chemical Engineering, Inner Mongolia University (Inner Mongolia), Hohhot 010021, China; 6 DICP-Surrey Joint Centre for Future Materials, Department of Chemical and Process Engineering, and Advanced Technology Institute, University of Surrey, Guildford GU2 7XH, Surrey, UK

**Keywords:** surface modification, hollow structure, charge separation, photocatalysis, H_2_ evolution

## Abstract

Engineering the surface structure of semiconductor is one of the most promising strategies for improving the separation and transfer efficiency of charge, which is a key issue in photocatalysis. Here, we designed and fabricated the C decorated hollow TiO_2_ photocatalysts (C–TiO_2_), in which 3-aminophenol-formaldehyde resin (APF) spheres were used as template and carbon precursor. It was determined that the C content can be easily controlled by calcinating the APF spheres with different time. Moreover, the synergetic effort between the optimal C content and the formed Ti–O–C bonds in C–TiO_2_ were determined to increase the light absorption and greatly promote the separation and transfer of charge in the photocatalytic reaction, which is verified from UV–vis, PL, photocurrent, and EIS characterizations. Remarkably, the activity of the C–TiO_2_ is 5.5-fold higher than that of TiO_2_ in H_2_ evolution. A feasible strategy for rational design and construction of surface-engineered hollow photocatalysts to improve the photocatalytic performance was provided in this study.

## 1. Introduction

With the development of human society and rampant industrial growth, the need for renewable hydrogen energy has become increasingly urgent due to the non-renewability of fossil fuels. Sunlight as an inexhaustible source of energy can be converted into electricity and chemical energy by catalysis technologies [1,2,3]. Photocatalytic H_2_ evolution from water splitting has attracted particular interest for the sustainable survival and development of mankind [4,5,6], whereupon semiconductors as photocatalysts are one of the most important and indispensable elements in photocatalytic reaction system [7]. In a wide variety of semiconductor catalysts, Titanium oxide (TiO_2_) as a promising photocatalyst has been widely studied for photocatalytic H_2_ production due to low toxicity and high stability [8,9,10]. Nevertheless, its photocatalytic application is still restricted owing to the rapid recombination of photogenerated charge carriers [11,12,13]. Consequently, it is highly desired to develop TiO_2_-based photocatalysts with superior photogenerated charge separation. 

A lot of strategies have been developed to enhance the photocatalytic performance, including the structural design and modification [11,12,13]. The fabrication of the hollow structure [14,15,16,17] has been extensively proved to increase the light absorption due to multiple reflection and refraction of light in the super-large cavity [18,19,20], and shorten the transfer distance of photogenerated charges [21]. In addition, element doping provides a unique opportunity to improve the photocatalytic performance by tuning the energy band and photogenerated charge separation efficiency [22,23]. For instance, Yang et al. reported that P doping efficiently improves photocatalytic H_2_ evolution of TiO_2_ photocatalyst by enhancing light harvesting and charge separation [24]. Reddy et al. prepared the N-doped TiO_2_ photocatalysts, and new levels of N atoms were generated near the valence band maximum of TiO_2_ to enhance the optical properties and increase the charge separation [25]. Incorporating dopants into the TiO_2_ not only effectively overcomes the drawback of low solar energy utilization capacity of pure TiO_2_, but also promotes the transfer of holes and photogenerated electrons. By affecting the electronic environment of O anion and Ti ion, the utilization of light under C doping was promoted and the sensitization of TiO_2_ was also increased. For instance, Zheng et al. reported that photocatalytic reactions were performed using light, and the performance of TiO_2_ toward H_2_ production was greatly improved by C doping [26].

Here, we fabricated the C modified hollow TiO_2_ photocatalysts (hollow C–TiO_2_) by hard-template method, in which the 3-aminophenol-formaldehyde resin (APF) nanospheres were selected as growth templates as well as carbon source for the hollow C–TiO_2_ photocatalysts. The hollow structure and the carbon content of the hollow C–TiO_2_ can be tunned by finely modulating the calcination conditions. Interestingly, the formation of Ti-O-C bonds on C–TiO_2_ photocatalysts via FTIR and XPS characterization resulted in a remarkable improvement for photocatalytic H_2_ evolution compared with bare TiO_2_. This enhancement is mainly due to improving separation and transfer of the charge, and it is verified by PL, SPV, and EIS characterizations. A common and effective method to construct the hollow photocatalysts with C doping for efficient photocatalytic H_2_ evolution is provided in this work.

## 2. Experimental

### 2.1. Materials

All reagents were used as received without further purification. 3-Aminophenol (3-AP, 99.0%) (Adamas Reagent Co., LTD, Shanghai, China). Tetrabutyl orthotitanate (TBOT, 98.0%) (Aladdin Co., LTD, Fengxian District, Shanghai, China). Ammonia aqueous solution (25.0–28.0%), absolute ethanol (EtOH, 99.7%), formaldehyde aqueous solution (37.0–40.0%), and acetonitrile (99.8%) were obtained from (Sinopharm Chemical Reagent Co. Ltd., Shanghai, China). Deionized (DI) water was used in the experiments.

Synthesis of the 3-aminophenol-formaldehyde resin spheres (APF spheres) and the carbon spheres (C spheres) was conducted. APF spheres were synthesized according to a previously reported procedure [27]. Typically, we mixed ammonia aqueous solution (0.2 mL) with a solution containing EtOH (16 mL) and deionized water (40 mL), then the mixed solution was stirred for 3 min. Subsequently, 3-AP (0.8 g) was added in the mixed solution and continually stirred for more than 30 min. Then, we added the formaldehyde aqueous solution (1.12 mL) to the above reaction solution and stirred for 24 h at 30 °C, and under a static condition the solution was subsequently heated at 100 °C for 24 h in a Teflon-lined autoclave. We recovered the solid product by centrifugation which was washed with ethanol three times. The resulting APF was heated for 2 h under N_2_ atmosphere at 700 °C to obtain pure carbon (C) sample.

Coating APF with TiO_2_ (APF@TiO_2_) was performed. The as-prepared APF (0.5 g) was dispersed in EtOH (100 mL) in an ice bath and vigorous stirring. Acetonitrile (35 mL) and ammonia aqueous solution (0.75 mL), TBOT (2 mL) in a mixed solvent of EtOH (15 mL) and acetonitrile (5 mL) were injected into the mixture. After 5 h, the composite was washed with ethanol and separated by centrifugation to obtain APF@TiO_2_ core–shell composites.

Synthesis of the C modified TiO_2_ photocatalysts (C–TiO_2_) was performed. The resulting APF@TiO_2_ core–shell composites were heated under air atmosphere at 450 °C with a heating rate of 5 °C/min, and maintained at 450 °C for 1 h, 2 h or 5 h to obtain APF-TiO_2_-x samples (x = 1, 2, 3, respectively). Then, the APF-TiO_2_-x composites were heated with a heating rate of 2 °C/min at 700 °C in N_2_ atmosphere for 2 h and cooled to room temperature, and the resulting samples were labelled, respectively, as C–TiO_2_–1, C–TiO_2_–2 and C–TiO_2_–3.

Synthesis of pure TiO_2_ was performed. Pure TiO_2_ was prepared under the above experimental conditions except for adding APF.

### 2.2. Characterization

The morphology features of the products were characterized using transmission electron microscope (TEM, HT7700, HHT Inc., Tokyo, Japan), scanning electron microscopy (SEM, FEI QUANTA 200 F, FEI inc., Hillsboro, NK, USA) and high-resolution transmission electron microscopy (HRTEM, JEM-F200, JEOL Inc., Tokyo, Japan)). The structural characteristics of catalyst were carried out by X-ray diffraction (XRD, D/Max2500PC (Rigaku Inc., Tokyo, Japan) analysis which used a Rigaku D/Max2500PC diffractometer. We used a home-assembled Raman spectrograph (DL-3 UV Raman spectroscopy with operando system) and excited the Raman spectra at 532 nm with spectral resolution of 2 cm^−1^. In the range of 4000–400 cm^−1^, a Nicolet Nexus 470 spectrometer using KBr pellet was used to record the Fourier-transform infrared (FTIR) spectra (Nicolet Inc., Mountain, WI, USA). We collected N_2_ adsorption–desorption isotherms on Micromeritics Tristar II 3020 automated analyzer at 77 K, and the surface areas were determined according to the Brunauer–Emmett–Teller (BET) method: Tristar II 3020 (Micromeritics Inc. Atlanta, GA, USA). X-ray photoelectron spectroscopy (XPS) was characterized on a Thermo Scientific^TM^ K-Alpha^TM+^ spectrometer (Thermo Scientific Inc., Waltham, MA, USA). We collected UV–vis diffuse reflectance spectra (DRS) on a UV-2700 spectrophotometer (SGLC Inc., Shanghai, China). Photoluminescence spectra (PL) (λex = 325 nm, λem = 340–600 nm) were measured using a JASCO fluorescence spectrometer (FP-8500) (JASCO China Inc., Shanghai, China) with a Xe lamp as the excitation light source at room temperature. Surface photovoltage (SPV) spectra were measured with a home-assembled surface photovoltage with a SR830 DSP lock-in amplifier of Stanford Research Systems at room temperature. TGA (Mettler-Toledo Inc., Zurich, Switzerland) analyses were performed on a TGA/DSC-1 thermogravimetric analyzer provided by the Mettler-Toledo Instrument.

### 2.3. Photoelectrochemical Measurements

The C–TiO_2_ samples (0.05 g) were dispersed in 2 mL of ethanol and 10 μL of 5% nafion reagent. Then, the above mixed solution was sonicated for 30 min. Finally, the given 50 μL solution was dropped on the conductive glass (FTO) and dried in order to use it as working electrodes. A platinum electrode was used as the counter electrode, and a saturated Ag/AgCl electrode was selected as the reference electrode. Electrochemical impedance spectroscopy (EIS) and the photocurrent-time curves of the samples were evaluated in 1 M Na_2_SO_4_ electrolyte in a three-electrode quartz cell, and performed by an electrochemical workstation (CH Instruments Inc, Shanghai, China). A 300w Xe lamp was used as the light source. Mott–Schottky (M-S) plots were measured in 0.1 M Na_2_SO_4_ electrolyte from 1 V to −1 V at 1000 Hz.

### 2.4. Photocatalytic Activity

Photocatalytic H_2_ evolution was carried out using a 300 W Xe lamp (prefect light) in a closed gas circulation and inhalation system. The 20.0 mg of the C–TiO_2_ photocatalyst was dispersed in solution containing H_2_O (80 mL) and CH_3_OH (20 mL), which was conducted by venting away the internal air to achieve complete degassing. We decided to evolve the amount of H_2_ by an online gas chromatograph (GC-7900, Shanghai Techcomp. TCD, Ar carrier, Shanghai, China).

## 3. Results and Discussion

**Figure 1:**
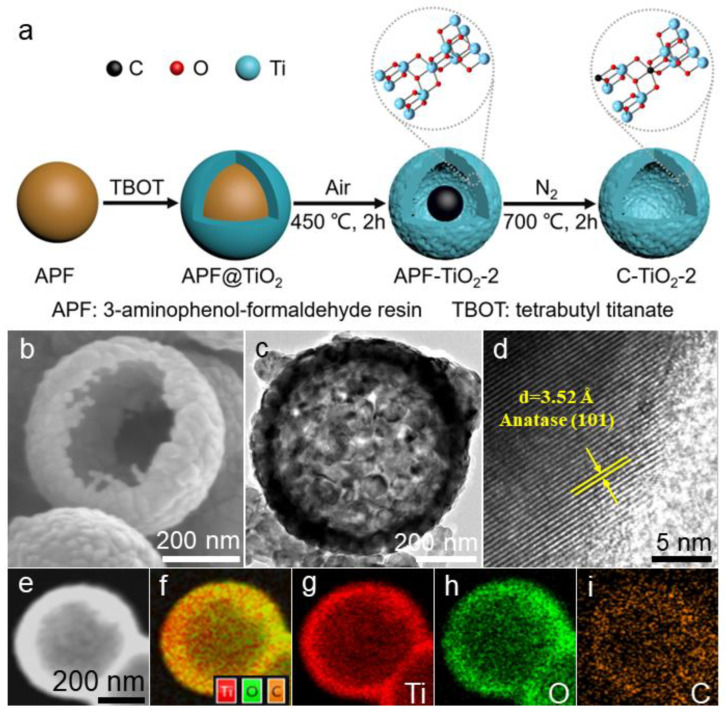
(**a**) Schematics showing the processes of synthesizing C-modified hollow photocatalysts (C–TiO_2_–2). (**b**–**d**) SEM, TEM and HRTEM images of the C–TiO_2_–2 photocatalyst. (**e**–**i**) EDS mapping images of C–TiO_2_–2 photocatalyst.

The schematic representation of the synthesis of the hollow C–TiO_2_ photocatalysts is presented in Figure 1a. The synthesis steps involved the preparation of 3-aminophenol-formaldehyde resin (APF) spheres templates (Figure S1), TiO_2_ coating on APF surface (APF@TiO_2_), and transformation of APF@TiO_2_ into hollow C–TiO_2_ photocatalysts through controlling the calcination conditions. Specifically, the TiO_x_ shells through the hydrolysis, oligomerization, and condensation of tetrabutyl orthotitanate (TBOT) coated on APF spheres surface to fabricate APF@TiO_2_ (Figure S2). The C–TiO_2_ hollow sphere was synthesized by heating APF@TiO_2_ in air atmosphere at 450 °C at different times to modulate the C content (APF-TiO_2_), followed by heating at 700 °C under N_2_ atmosphere to carbonize APF. The resulting hollow C–TiO_2_ samples were labelled as C–TiO_2_–1, C–TiO_2_–2 and C–TiO_2_–3, respectively. SEM images of a representative sample, C–TiO_2_–2, show uniform microsphere morphologies with a diameter of approximately 750 nm (Figure S3), among which a broken sphere (Figure 1b) clearly indicates the hollow sphere structure. As shown in Figure 1c, the hollow microsphere is uniform and regular. The average particle sizes calculated by TEM images were 750 nm, which is in agreement with SEM results. HRTEM further investigated the crystal structure of the synthesized C–TiO_2_–2 (Figure 1d). It can be seen that the lattice spacing of C–TiO_2_–2 is 0.35 nm, which can be well directed to the (101) flat of anatase type TiO_2_ [28]. The EDS elemental mapping of the energy spectrum in Figure 1f–i, confirms that Ti, O, and C are distributed very uniformly in the hollow spheres of C–TiO_2_–2, indicating the successful incorporation of C.

**Figure 2:**
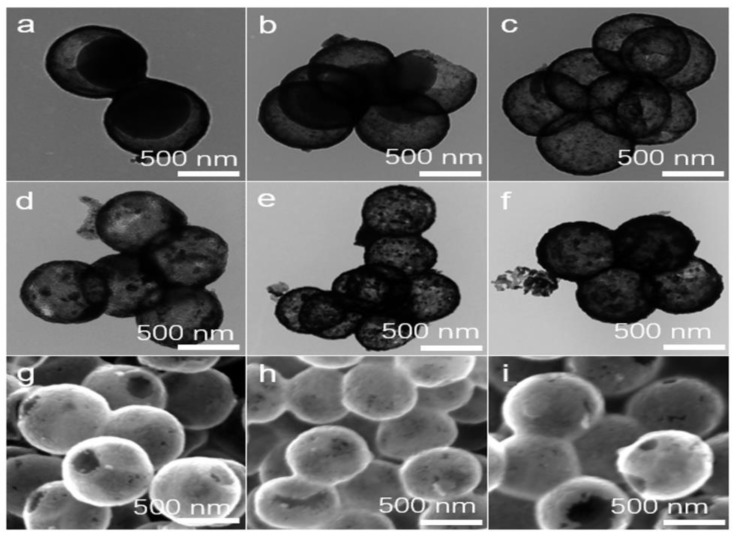
(**a**–**c**) TEM images of the obtained APF–TiO_2_–1, APF–TiO_2_–2 and APF–TiO_2_–3 photocatalysts under air calcining. (**d**–**f**) TEM images of the obtained C–TiO_2_–1, C–TiO_2_–2 and C–TiO_2_–3 under N_2_ carbonization for 2 h and (**g**–**i**) the corresponding SEM images. The scale bar is 500 nm.

To clarify the formation process of hollow C–TiO_2_ photocatalysts, several samples obtained by controlling the calcination condition are described in detail. Figure 2a–c displays the morphological evolution of APF@TiO_2_ calcined at 450 °C under air atmosphere at different durations. Yolk–shell APF@TiO_2_ sphere and hollow APF@TiO_2_ sphere could be fabricated at a calcination of 1 h and 5 h, respectively. All the C–TiO_2_ samples by carbonization APF@TiO_2_ samples at 700 °C for 2 h under N_2_ atmosphere show the monodisperse nanospheres (Figure 2g–i) with hollow structure (Figure 2d–f).Notably, the C contents estimated by TGA method were approximately 9.1 wt.%, 0.65 wt.%, and 0.085 wt.% for C–TiO_2_–1, C–TiO_2_–2, and C–TiO_2_–3, respectively, indicating that the C contents in the C–TiO_2_ photocatalyst can be easily tuned by altering the calcination conditions (Table S1). As shown in Figure S4, all the C–TiO_2_–1, C–TiO_2_–2 and C–TiO_2_–3 samples exhibit type IV N_2_ adsorption–desorption isotherms with a H1 hysteresis loop, which corresponds to the microporous structure. Table S2 shows that the specific surface area of C–TiO_2_–1, C–TiO_2_–2 and C–TiO_2_–3 is 98, 19 and 14 m^2^/g, respectively, proving that the C content has significant effect on the specific surface area of the hollow C–TiO_2_.

**Figure 3:**
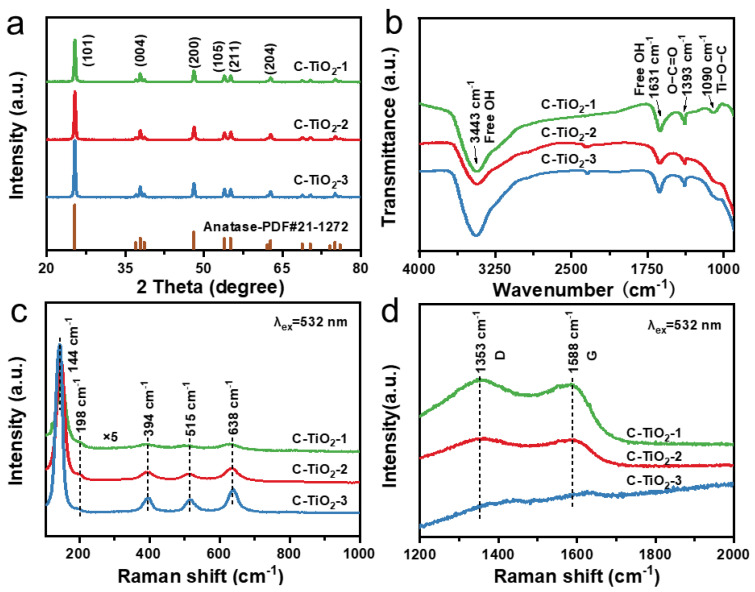
(**a**) Powder XRD patterns of the obtained C–TiO_2_–1, C–TiO_2_–2 and C–TiO_2_–3 photocatalysts. (**b**) FTIR spectra of the corresponding samples. (**c**,**d**) Raman spectra of the corresponding samples.

Furthermore, the structure and composition of C–TiO_2_ samples were studied by XRD, FTIR and Raman spectroscopy. As shown in Figure 3a, the main diffraction peaks (25.3°, 37.8°, 48.1°, 53.9°, 55.1° and 62.7°) of all the hollow C–TiO_2_ samples can be indicated as anatase phase. They correspond to (101), (004), (200), (105), (211) and (204) crystal flats. (JCPDS, No. 21–1272). Nevertheless, the peaks increased in intensity with rising of the annealing time under air atmosphere, which is primarily due to the reduced shielding impact of carbonaceous species in the TiO_2_ hollow structures as well as the grain growth of TiO_2_ [29]. FTIR spectroscopy also confirmed successful introduction of C into TiO_2_, with characteristic bands of C appearing at ∼1393 cm^−1^ corresponding to O–C=O vibrations (Figure 3b). Furthermore, compared with pure TiO_2_, an extra FTIR band at 1090 cm^−1^ in connection with Ti–O–C stretching is observed for all the hollow C–TiO_2_ samples, indicating that Ti–O–C covalent bonds are formed in the C–TiO_2_ (Figure S5) [30].

The Raman spectra of hollow C–TiO_2_ photocatalysts with the excitation line at 532 nm are shown in Figure 3c,d. For all the C–TiO_2_ samples, Raman bands at 144, 198, 394, 515, and 638 cm^−1^ are observed, which is ascribed to the E_g_, E_g_, B_1g_, A_1g_ + B_1g_, and E_g_ modes of anatase phase TiO_2_. Obviously, the stronger Raman bands in intensity are observed for the C–TiO_2_–3 sample compared with C–TiO_2_–1 and C–TiO_2_–2, suggesting that C–TiO_2_–3 sample displays higher crystallinity. The results are consistent with the XRD. Furthermore, the characteristic Raman bands of the D and G vibration modes of C at 1353 cm^−1^ and 1588 cm^−1^ are obviously observed in the C–TiO_2_–1, indicating that the C element is incorporated into hollow TiO_2_ photocatalyst. However, the D and G bands decreased in intensity in the C–TiO_2_–2 sample, while these two Raman bands disappeared nearly in the C–TiO_2_–3 sample, suggesting that the C content gradually decreased when the calcination time for APF@TiO_2_ under air atmosphere was increased from 1 h to 5 h, which is consistent with that from Table S1.

**Figure 4:**
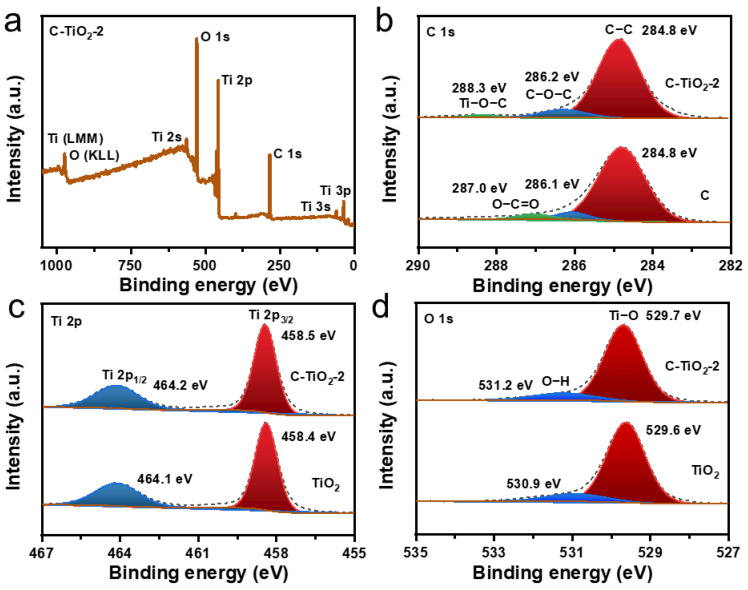
(**a**) XPS survey spectra of the prepared C–TiO_2_–2. (**b**) C 1s XPS spectra of the C and C–TiO_2_–2. (**c**) Ti 2p XPS spectra of the TiO_2_ and C–TiO_2_–2. (**d**) O 1s XPS spectra of the TiO_2_ and C–TiO_2_–2.

More detailed information regarding chemical properties of pure carbon spheres (denoted as C) formed by calcination of APF and the hollow C–TiO_2_ photocatalyst were obtained by XPS. On the basis of the XPS spectroscopic analysis, it was determined that Ti, O and C existed in the hollow C–TiO_2_–2 photocatalyst (Figure 4a). In the high resolution C1s XPS of hollow C–TiO_2_–2 photocatalyst (Figure 4b), the fitted peaks centred at 284.8, 286.1, and 287.0 eV, which were ascribed to the sp^2^ hybridized C (C–C bonds), the oxygen-bound substance C–O and C=O bonds [31,32,33]. Compared with pure C spheres, the emerging peak in C–TiO_2_–2 at 288.3 eV is ascribed to the formation of the Ti–O–C covalent band [34]. In Figure 4c, the high-resolution Ti 2p spectra, the two peaks centring at 458.5 eV and 464.2 eV were due to Ti 2p_3/2_ and Ti 2p_1/2_. It is reported that C enters the lattice and generates a Ti–O–C covalent bonds and hybrid orbital is formed above the valence band, which enhances visible light adsorption capacity of TiO_2_ [35]. Compared with pure TiO_2_, the characteristic peak of C–TiO_2_–2 at 458.4 eV occurs blue-shifted, which could be related to the change in the environment around Ti^4+^ species in carbon-doped sample. As shown in Figure 4d, the O1s peak can be decomposed into two peaks, in which the peak at 529.7 eV can be ascribed to lattice oxygen in oxides, and the peak at 531.2 eV was ascribed to hydroxyl (-OH) group [36]. At the same time, we also conducted XPS for C–TiO_2_–1 and C–TiO_2_–3 samples (Figure S11).

**Figure 5:**
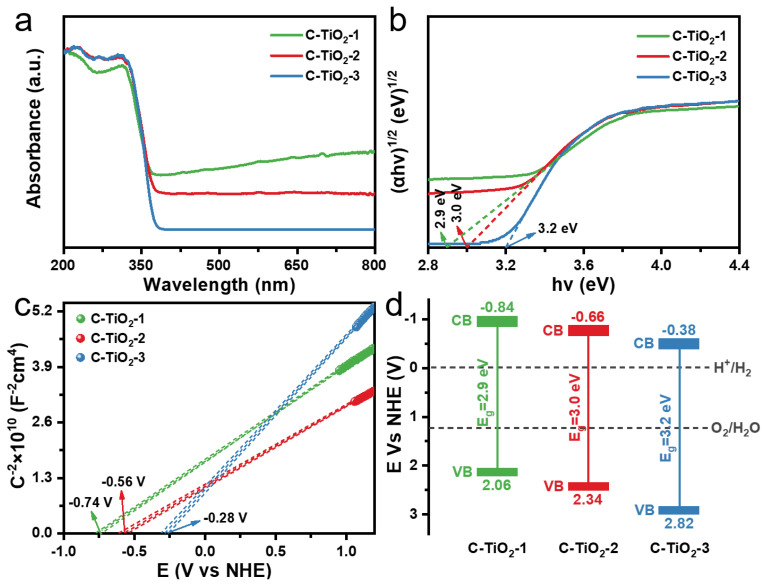
(**a**,**b**) Diffuse reflectance spectrum of the prepared C–TiO_2_–1, C–TiO_2_–2 and C–TiO_2_–3 photocatalysts and Tauc plots. (**c**) Mott–Schottky plots and (**d**) Electronic band diagram of the C–TiO_2_–1, C–TiO_2_–2 and C–TiO_2_–3.

DRS and M-S measurements were used to study the optical absorption characteristics and electronic bands of the hollow C–TiO_2_ samples. As displayed in Figure 5a, all the hollow C–TiO_2_ samples show similar light absorbance in the range of 200–390 nm. In the visible region (400–800 nm), the C–TiO_2_–1 sample shows the strong absorption due to its high C content, while C–TiO_2_–3 does not absorb the visible light, which could be ascribed to its very low C content. We can calculate the band gap by (ahυ)^1/2^ curves of the photon energy (hυ) and the band gap energy (E_g_) of C–TiO_2_–1, C–TiO_2_–2 and C–TiO_2_–3 is calculated to be 2.9, 3.0 and 3.2 eV (Figure 5b). The flat-band energy potential (E_FB_) of the C–TiO_2_ samples was investigated by M-S measurements. We calculated E_FB_ values using the intercept of axes with potential values at −0.74, −0.56 and −0.28 V vs. NHE for C–TiO_2_–1, C–TiO_2_–2 and C–TiO_2_–3, respectively (Figure 5c). E_FB_ is regarded as approximately 0.1 V below the conduction band (E_CB_) in many n-type semiconductors [37]. The E_CB_ for C–TiO_2_–1, C–TiO_2_–2 and C–TiO_2_–3 were figured up to be −0.84, −0.66 and −0.38 V, vs. NHE (Figure 5d). Obviously, the hollow TiO_2_ with Ti-O-C bond resulted in the change in the electronic energy band, and the energy band structure of C–TiO_2_ can be easily adjusted by carbon doping amount.

**Figure 6:**
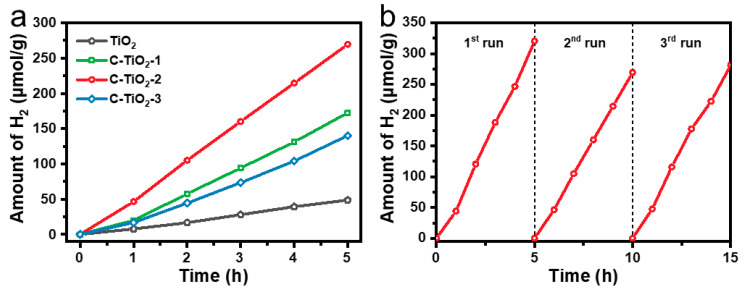
(**a**) Photocatalytic hydrogen evolution under simulated solar light irradiation over different samples. (**b**) Cycling tests of photocatalytic hydrogen evolution of the C–TiO_2_–2 photocatalysts under simulated solar light irradiation (methanol as a sacrificial agent).

To emphasize the important role of surface modification on TiO_2_ photocatalytic activity, the photocatalytic hydrogen production of C–TiO_2_ photocatalysts was evaluated in water/methanol solution under Xe lamp irradiation. The pure TiO_2_ showed low photocatalytic activity, while the C incorporation in TiO_2_ greatly increased the photocatalytic H_2_ evolution (Figure 6a and Figure S12). Moreover, the photocatalytic activity of hollow C–TiO_2_ is relevant to the C content, where C–TiO_2_–2 showed the highest activity and reached 5.5 times higher than that of pure TiO_2_. Furthermore, the specific H_2_ evolution rates (the overall H_2_ evolution rate was divided by the surface area) of C–TiO_2_ and TiO_2_ were also compared (Figure S6). Obviously, the specific H_2_ evolution rates for C–TiO_2_ exhibited similar trend to the overall H_2_ production rate. These results indicate that surface area is not the main factor to enhance of C–TiO_2_–2 photocatalytic activity. Though the C–TiO_2_–2 exhibits the weaker light absorption intensity in the visible region compared to C–TiO_2_–1, the remarkable enhancement for the photocatalytic H_2_ evolution rate highlights the importance of the optimal C content in the hollow C–TiO_2_ in photocatalysis. In the stability test, there was no serious inactivation for the C–TiO_2_–2 in the measurement process of 15 h (Figure 6b). Moreover, the C–TiO_2_–2 after the photocatalytic experiments were further characterized by SEM, XRD, Raman and XPS (Figures S7–S10). The hollow structure and crystal phase of the C–TiO_2_–2 were well preserved, suggesting that the C–TiO_2_ photocatalysts for photocatalytic activity exhibit good long-term durability.

**Figure 7:**
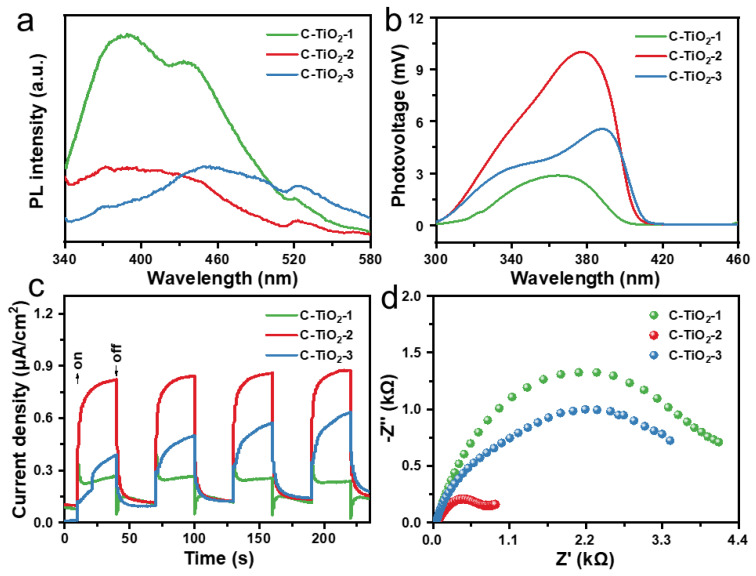
(**a**) Photoluminescence spectra. (**b**) Surface photovoltage (SPV) amplitude spectra at the nanoscale level under illumination from 300 to 460 nm. (**c**) Photocurrent responses and (**d**) Nyquist plots of as-prepared samples under light irradiation.

**Figure 8:**
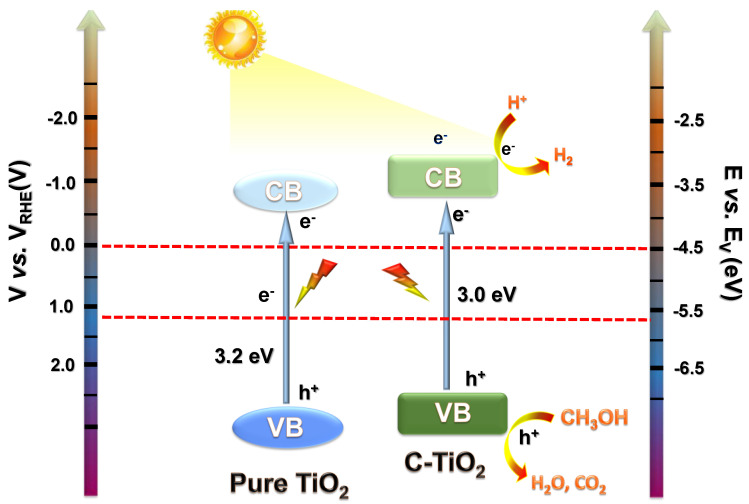
Schematic illustration for the photocatalytic H_2_ production mechanism over the C–TiO_2_ sample under light irradiation.

To investigate the main reason for the strong photocatalytic performance on the C–TiO_2_–2, a series of characterizations were used. PL spectra were recorded to characterize the recombination efficiency of photogenerated electrons and holes from the C–TiO_2_ (Figure 7a). C–TiO_2_–1 exhibits a strong PL intensity, which is caused by the reorganization of the native carriers. C–TiO_2_–3 has a stronger trapping ability and a stronger carrier separation ability compared with pure C–TiO_2_–1. The PL intensity of C–TiO_2_–2 is significantly quenched compared with C–TiO_2_–1 and C–TiO_2_–3, implying that optimized C content is beneficial for charge separation. Moreover, under light irradiation, SPV spectroscopy was used to reveal the change in surface potential barrier of C–TiO_2_ samples (Figure 7b). Notably, the SPV signal of C–TiO_2_–2 is much stronger than that of the C–TiO_2_–1 and C–TiO_2_–3, which means that the separation of electrons and holes is greatly promoted in C–TiO_2_–2. As displayed in Figure 7c, the instantaneous photocurrent response results show that C–TiO_2_–2 have the highest photocurrent density compared to C–TiO_2_–1 and C–TiO_2_–3, indicating better charge separation and transfer ability of C–TiO_2_–2. The charge transfer resistance tested from the EIS with the fitted circuit diagram is displayed in Figure 7d. The results show that the smaller the radius, the smaller the interface charge transfer resistance. Obviously, in the EIS Nyquist plots, the arc radius of the C–TiO_2_–2 photocatalyst is smaller than that of the C–TiO_2_–1 and C–TiO_2_–3, explaining that the interfacial transfer charge resistance between the electrolyte and electrode of the C–TiO_2_–2 photocatalyst is much smaller [38]. These results demonstrate that the superior photocatalytic activity on C–TiO_2_–2 can be attributed to C modification as well as hollow TiO_2_ structure, promoting the charge separation and transfer ability. As shown in Figure 8, the mechanism of H_2_ generation by C–TiO_2_ photocatalysis is as follows: Photogenerated electron–hole pairs are generated from C–TiO_2_ under photoexcitation conditions. Then, CH_3_OH is used as sacrificial reagent to react with h^+^, and the e^-^ reduces H^+^ to produce H_2_. Our work reports high activity compared to other related types of work at the same time (Table S3).

## 4. Conclusions

In summary, we constructed C-modified hollow TiO_2_ photocatalysts by calcinating APF spheres as template and carbon source. The formed Ti–O–C bonds on the surface of TiO_2_ shell are confirmed by FTIR and XPS spectra, which is of benefit to promote the fact that the photoexcited charge carriers separate and transfer. C-modified TiO_2_ exhibited improved performance for photocatalytic H_2_ evolution. Remarkably, the H_2_ evolution activity of C–TiO_2_–2 is 5.5-fold higher than that of bare TiO_2_. We believe that our study provides a typical case for the efficient conversion of solar energy by hollow photocatalysts.

## Data Availability

The original contributions presented in the study are included in the article/supplementary material, further inquiries can be directed to the corresponding author.

## Supplementary Materials

The following supporting information can be downloaded at: https://www.mdpi.com/article/10.3390/nano13050926/s1. Figure S1. SEM images of APF at different magnifications (a) 2 μm (b) 200 nm. Figure S2. SEM images of APF@TiO_2_ at different magnifications (a) 2 μm (b) 1 μm. Figure S3. TEM images of the (a) APF–TiO_2_–2 and (b) C–TiO_2_–2. Figure S4. Nitrogen adsorption-desorption isotherms of the prepared C–TiO_2_–1, C–TiO_2_–2 and C–TiO_2_–3 nanoreactors. Figure S5. FTIR spectrum of pure C sphere. Figure S6. Photocatalytic hydrogen evolution of pure TiO_2_, C–TiO_2_–1, C–TiO_2_–2 and C–TiO_2_–3 with normalized by their specific surface area separately under simulated solar light irradiation. Figure S7. SEM images of the C–TiO_2_–2 before (a) and after (b) three cycles of photocatalytic hydrogen evolution. Figure S8. XRD patterns of the C–TiO_2_–2 before and after three cycles of photocatalytic hydrogen evolution. Figure S9. (a) Raman spectra of C–TiO_2_–2 sample before and after three cycles of photocatalytic hydrogen evolution. (b) Partial enlargement of the selective area in (a). Figure S10. (a) XPS spectra of the C–TiO_2_–2 before and after three cycles of photocatalytic hydrogen evolution.(b–d) C 1s, Ti 2p, O 1s XPS spectra of the C–TiO_2_–2 before and after three cycles of photocatalytic hydrogen evolution. Figure S11. (a) XPS survey spectra of the prepared C–TiO_2_–1 and C–TiO_2_–3. (b) C 1s XPS spectra of the C–TiO_2_–1 and C–TiO_2_–3. (c) Ti 2p XPS spectra of the C–TiO_2_–1 and C–TiO_2_–3. (d) O 1s XPS spectra of the C–TiO_2_–1 and C–TiO_2_–3. Figure S12. Photocatalytic hydrogen evolution of sample C–TiO_2_–2 under simulated sunlight irradiation. Table S1. The estimated carbon content in C–TiO_2_–1, C–TiO_2_–2 and C–TiO_2_–3 by TGA method. Table S2. Summary parameters of related sample from the N_2_ adsorption dates. Table S3. Comparison of similar jobs [26,39,40,41,42,43,44].
