# Supplementary material for: Surface Modification of Hollow Structure TiO2 Nanospheres for Enhanced Photocatalytic Hydrogen Evolution
Source: Nanomaterials (Basel). 2023 Mar 3;13(5):926. doi: 10.3390/nano13050926 (PMC10004735; doi:10.3390/nano13050926)
Supplement: Supplementary file 1 [file nanomaterials-13-00926-s001.zip › nanomaterials-2167522-SI.pdf]

nanomaterials

MDPI

Supplementary Material

Surface Modification of Hollow Structure TiO_2 Nanospheres for Enhanced Photocatalytic Hydrogen Evolution

Gaomin Ning [1,†], Yan Zhang [2,†], Chunjing Shi [3,4,†], Chen Zhao [3,4], Mengmeng Liu [2], Fangfang Chang [3,4], Wenlong Gao [1], Sheng Ye [2], Jian Liu [3,4,5,6,*] and Jing Zhang [1,*]

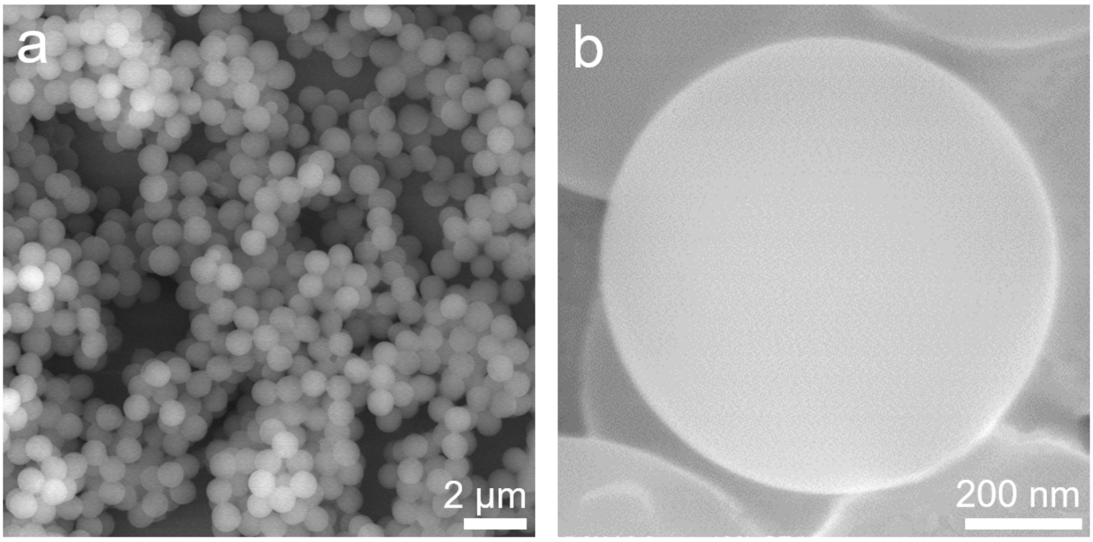

Figure S1. SEM images of APF at different magnifications **(a)** 2 μm **(b)** 200 nm.

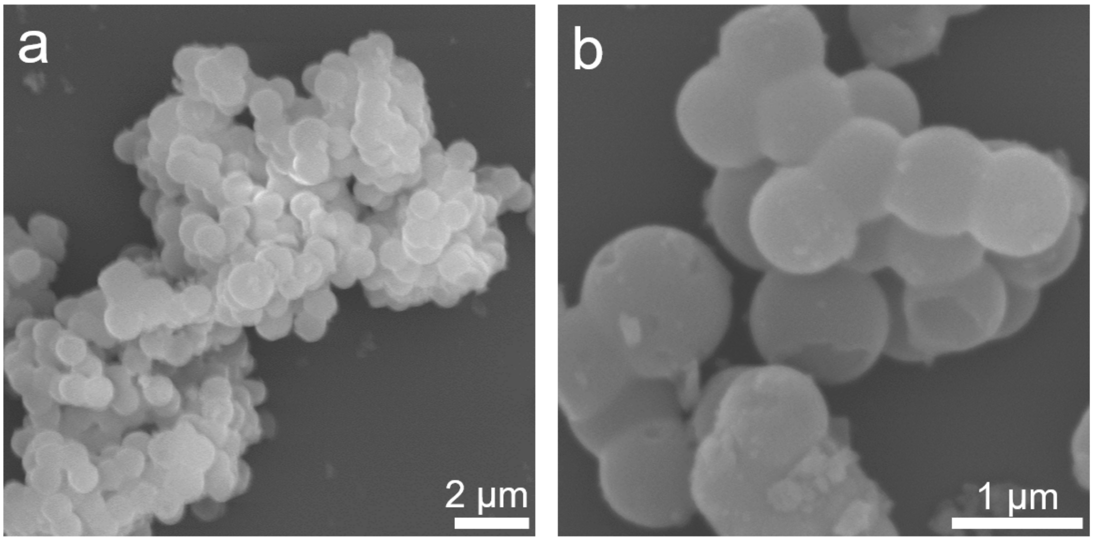

Figure S2. SEM images of APF@TiO_2 at different magnifications **(a)** 2 μm **(b)** 1 μm .

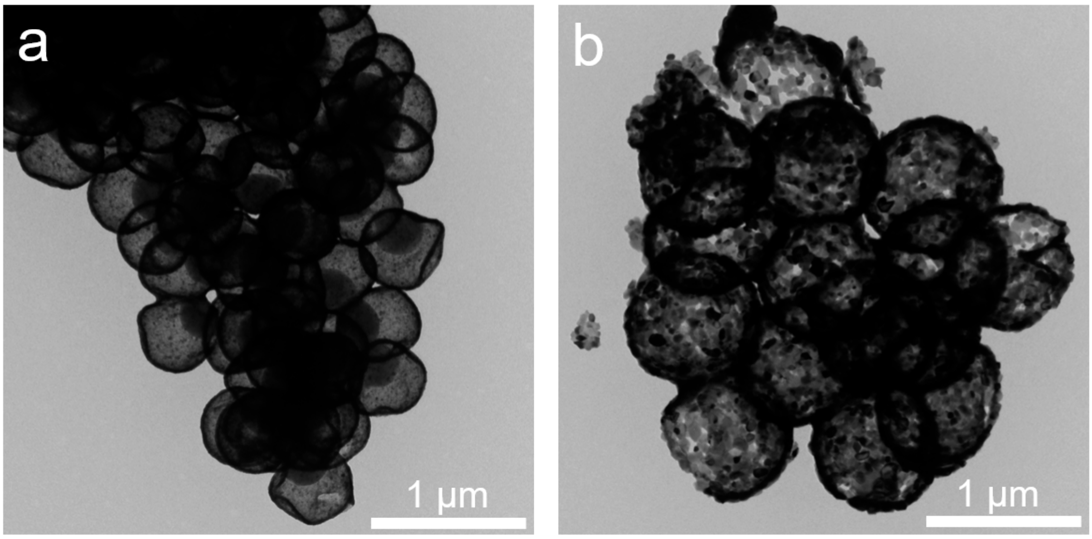

Figure S3. TEM images of the **(a)** APF−TiO_2−2 and **(b)** C−TiO_2−2.

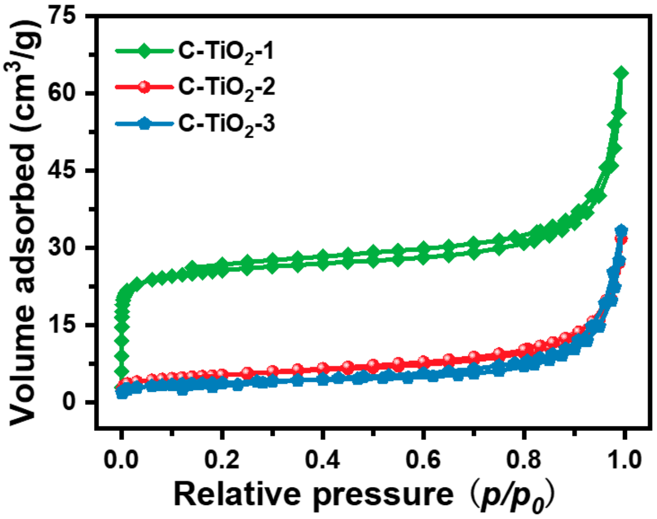

Figure S4. Nitrogen adsorption-desorption isotherms of the prepared C−TiO_2−1, C−TiO_2−2 and C−TiO_2−3 nanoreactors.

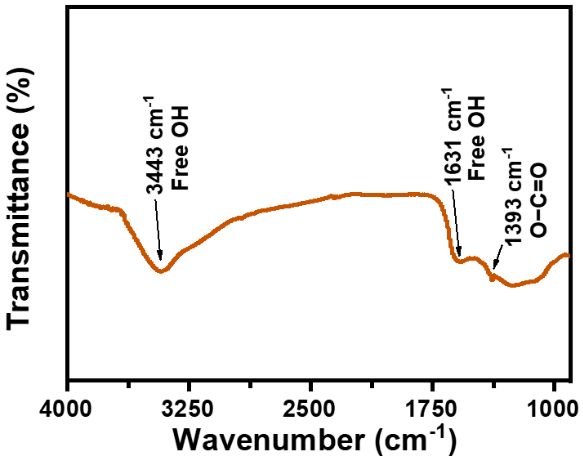

Figure S5. FTIR spectrum of pure C sphere.

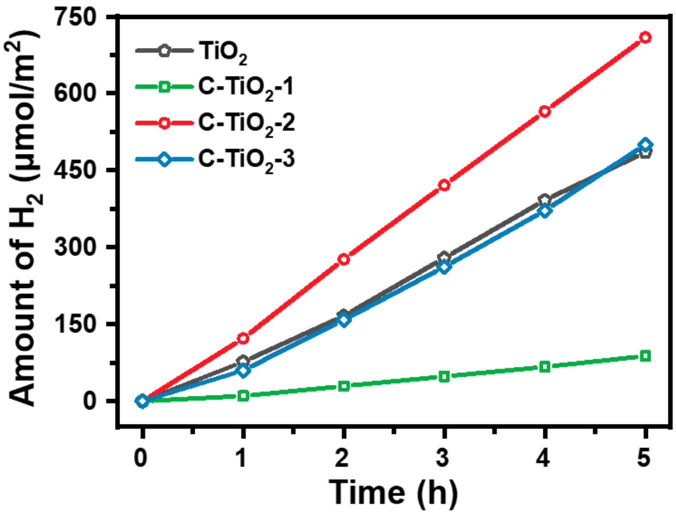

Figure S6. Photocatalytic hydrogen evolution of pure TiO_2, C−TiO_2−1, C−TiO_2−2 and C−TiO_2−3 with normalized by their specific surface area separately under simulated solar light irradiation.

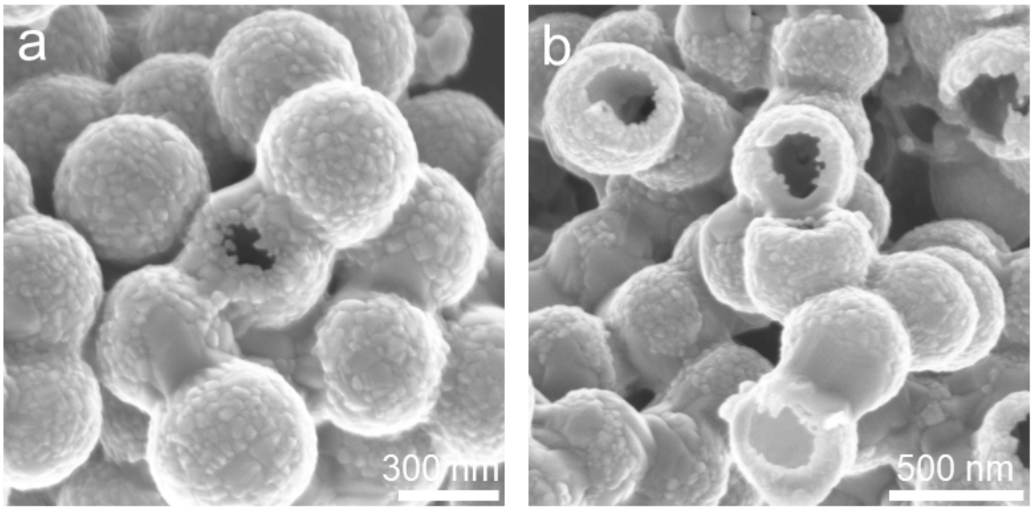

Figure S7. SEM images of the C−TiO_2−2 before **(a)** and after **(b)** three cycles of photocatalytic hydrogen evolution.

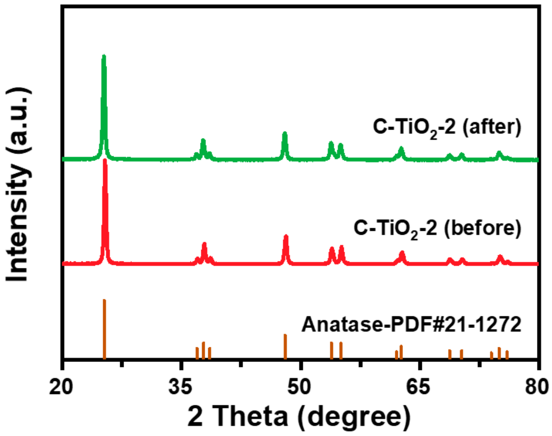

Figure S8. XRD patterns of the C−TiO_2−2 before and after three cycles of photocatalytic hydrogen evolution.

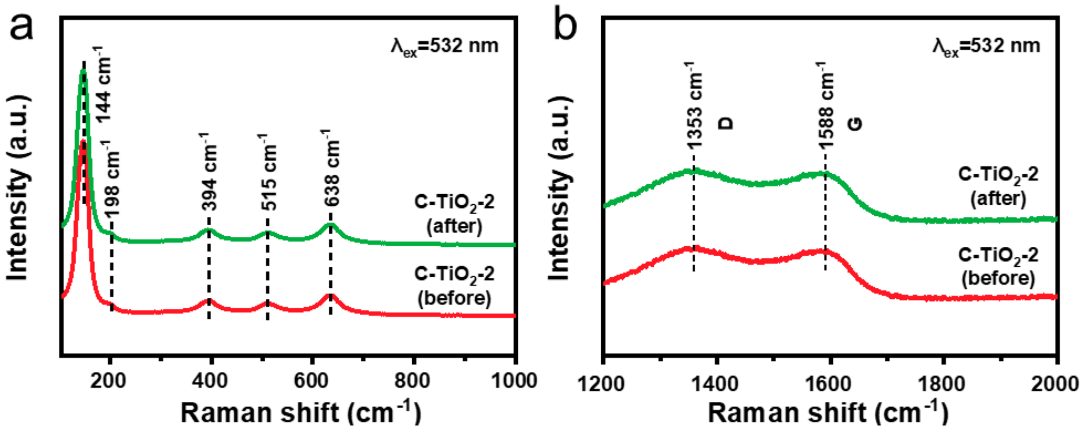

Figure S9. (a) Raman spectra of C−TiO2−2 sample before and after three cycles of photocatalytic hydrogen evolution. **(b)** Partial enlargement of the selective area in (a).

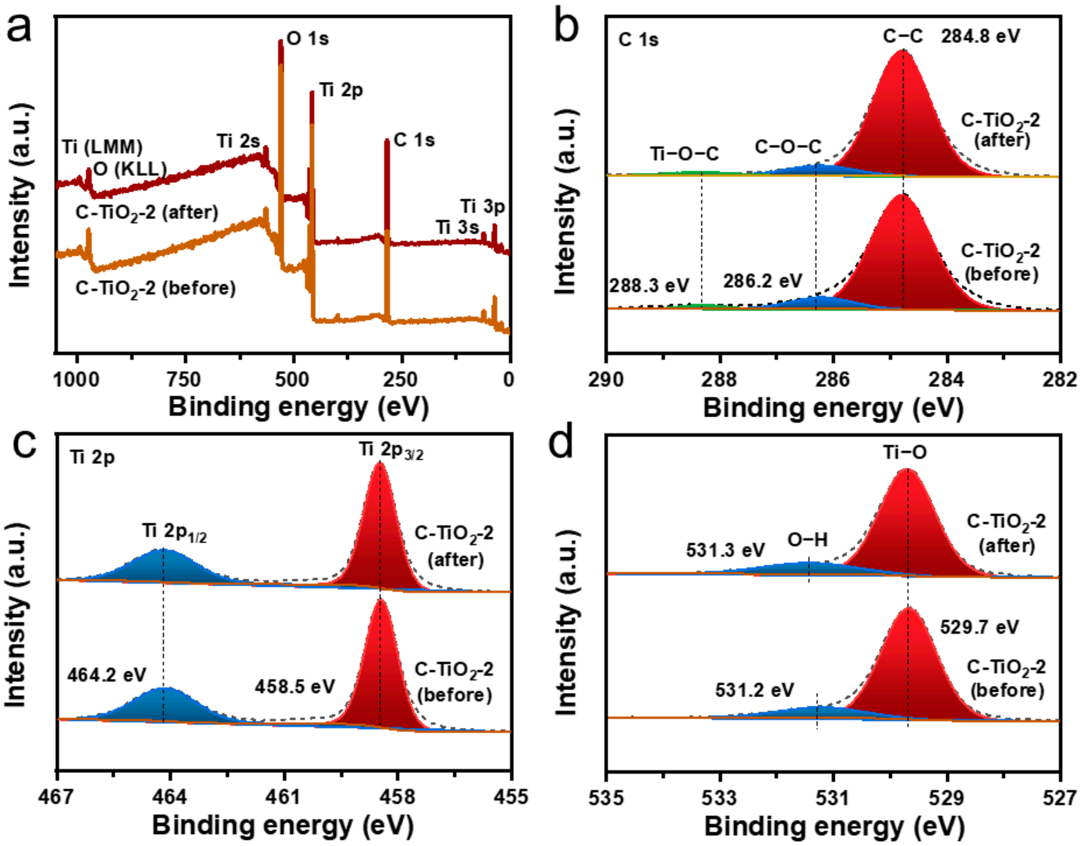

Figure S10. (a) XPS spectra of the C−TiO2−2 before and after three cycles of photocatalytic hydrogen evolution.**(b–d)** C 1s, Ti 2p, O 1s XPS spectra of the C−TiO2−2 before and after three cycles of photocatalytic hydrogen evolution.

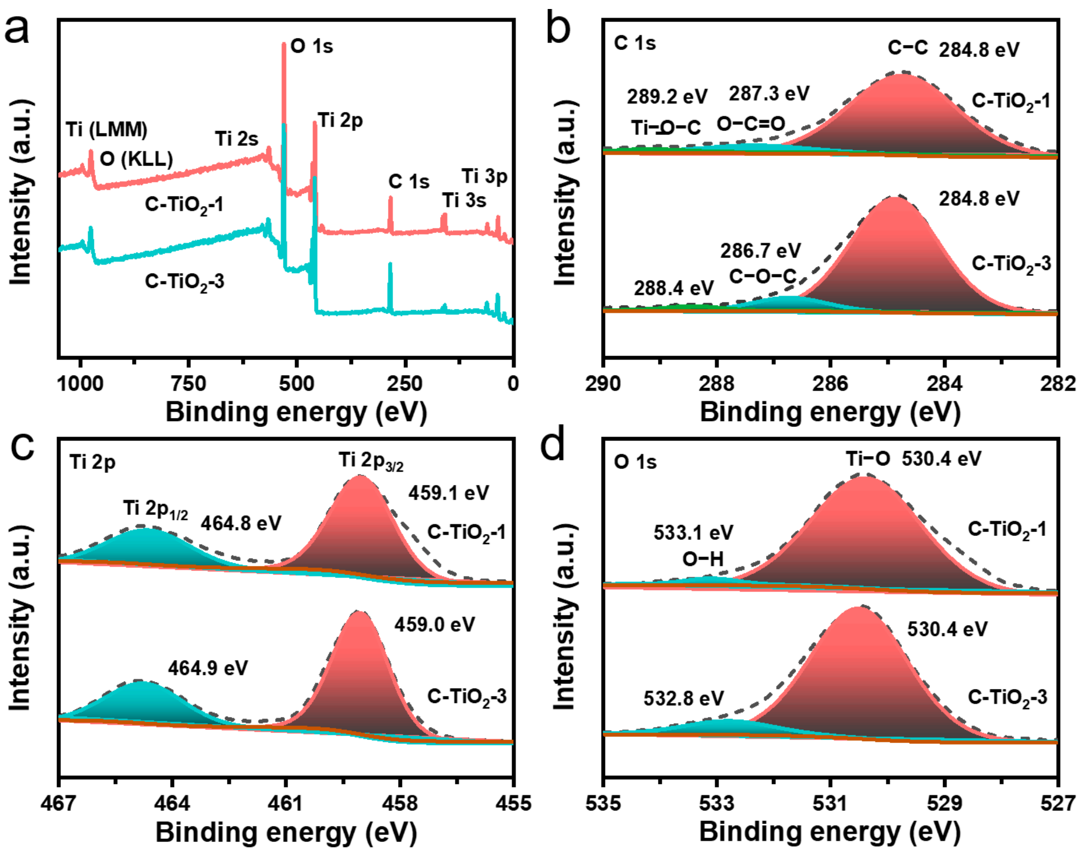

Figure S11. **(a)** XPS survey spectra of the prepared C–TiO_2–1 and C–TiO_2–3. **(b)** C 1s XPS spectra of the C–TiO_2–1 and C–TiO_2–3. **(c)** Ti 2p XPS spectra of the C–TiO_2–1 and C–TiO_2–3. **(d)** O 1s XPS spectra of the C–TiO_2–1 and C–TiO_2–3.

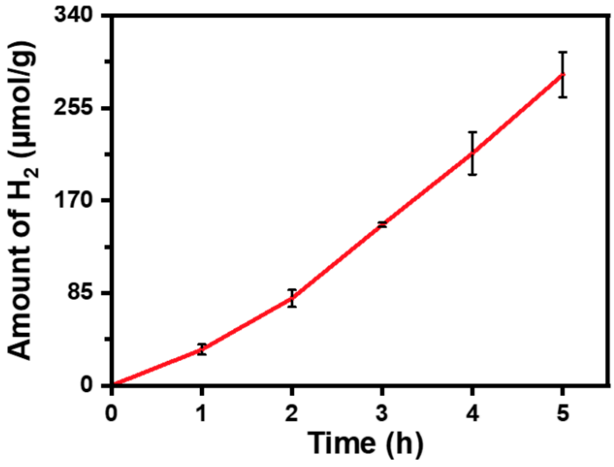

Figure S12. Photocatalytic hydrogen evolution of sample C–TiO_2–2 under simulated sunlight irradiation.

Table S1. The estimated carbon content in C–TiO_2–1, C–TiO_2–2 and C–TiO_2–3 by TGA method.

Sample	C-TiO_2-1	C-TiO_2-2	C-TiO_2-3
C/wt.%	9.11	0.65	0.085

Table S2. Summary parameters of related sample from the N_2 adsorption dates.

Sample	S_{BET} (m²/g)	Pore volume (cm³/g)	Average pore size (nm)
TiO_2	5	0.106	8.11
C-TiO_2-1	98	0.066	15.25
C-TiO_2-2	19	0.045	15.73
C-TiO_2-3	14	0.048	26.45

Table S3. Comparison of similar jobs.

Entry	Systems	Conditions	Activity (μmol/h/g)	Light source	Reference
1	C-TiO_2-2	Water-methanol	55	300 W Xe lamp	Our work
2	HC-TiO_2	Water-TEOA	33	300 W Xe lamp	[1]
3	TiO_2 nanobelt	Water-ethanol	~22	300 W Xe lamp	[2]
4	TiO_2 (N0)	Water-methanol	~14	UV-LEDs, 365 nm	[3]
5	TiO_2 (P25)	Water-methanol	30	350 W Xe lamp	[4]
6	0.1% Yb^{3+}-doped TiO_2	Water-TEOA	74	300 W Xe lamp	[5]
7	Comm. TiO_2 (P-25)	Water-methanol	82	500 W Xe/Hg lamp	[6]
8	TiO_2 nanofiber	Water-methanol	~74	400 W Hg lamp	[7]

References

1. Jia, G.; Wang, Y.; Cui, X.; Zheng, W. Highly Carbon-doped TiO_2 Derived from MXene Boosting the Photocatalytic Hydrogen Evolution. *ACS. Sustain. Chem. Eng.* **2018**, *6*, 13480–13486.
2. Yi, L.; Lan, F.; Li, J.; Zhao, C. Efficient noble-metal-free Co-NG/TiO_2 photocatalyst for H_2 evolution: Synergistic effect between single-atom Co and N-doped graphene for enhanced photocatalytic activity. *ACS. Sustain. Chem. Eng.* **2018**, *10*, 12766–12775.
3. Yu, J.; Hai, Y.; Cheng, B. Enhanced Photocatalytic H_2-Production Activity of TiO_2 by $Ni(OH)_2$ Cluster Modification. *J. Phys. Chem. C*, **2011**, *11*, 4953–4958.
4. Si, J.; Wang, Y.; Xia, X.; Peng, S.; Wang, Y.; Xiao, S.; Gao, Y. Novel quantum dot and nano-sheet TiO_2 (B) composite for enhanced photocatalytic H_2 – Production without Co-Catalyst. *Jops*, **2017**, *360*, 353–359.
5. Zhu, Y.; Zhang, Z.; Lu, N.; Hua, R.; Dong, B. Prolonging charge-separation states by doping lanthanide-ions into {001}/{101} facets-coexposed TiO_2 nanosheets for enhancing photocatalytic H_2 evolution. *Chin. J. Cata.* **2019**, *3*, 413–423.
6. Zahid Hussain, M.; Yang, Z.; Van der Linden, B.; Huang, Z.; Jia, Q.; Cerrato, E.; Xia, Y. Surface functionalized N-C-TiO_2/C nanocomposites derived from metal-organic framework in water vapour for enhanced photocatalytic H_2 generation. *J. Energy. Chem.* **2021**, 57,485–495.
7. Bai, H.; Liu, Z.; Sun, D.D. Facile Fabrication of TiO_2/$SrTiO_3$ Composite Nanofibers by Electrospinning for High Efficient H_2 Generation, *J. Am. Ceram. Soc.* **2012**, *3*, 942–949.
